# Carbapenem-Resistant Enterobacteriaceae—Implications for Treating Acute Leukemias, a Subgroup of Hematological Malignancies

**DOI:** 10.3390/antibiotics10030322

**Published:** 2021-03-19

**Authors:** Kristin Ølfarnes Storhaug, Dag Harald Skutlaberg, Bent Are Hansen, Håkon Reikvam, Øystein Wendelbo

**Affiliations:** 1 Department of Medicine, Stord Hospital, 5416 Stord, Norway; kristin.storhaug@live.com; 2 Department of Microbiology, Haukeland University Hospital, 5021 Bergen, Norway; dag.harald.skutlaberg@helse-bergen.no; 3 Department of Clinical Science, Faculty of Medicine, University of Bergen, 5020 Bergen, Norway; Hakon.Reikvam@uib.no; 4 Department of Medicine, Førde Hospital, 6812 Førde, Norway; bent-are.hansen@helse-forde.no; 5 Department of Medicine, Haukeland University Hospital, 5021 Bergen, Norway; 6 Faculty of Health, VID Specialized University, 5020 Bergen, Norway; 7 Department of Cardiology, Haukeland University Hospital, 5021 Bergen, Norway

**Keywords:** carbapenem-resistant Enterobacteriaceae, infections, acute leukemia

## Abstract

Acute leukemias (AL) are a group of aggressive malignant diseases associated with a high degree of morbidity and mortality. Patients with AL are highly susceptible to infectious diseases due to the disease itself, factors attributed to treatment, and specific individual risk factors. Enterobacteriaceae presence (e.g., *Klebsiella pneumonia* and *Escherichia coli)* is a frequent cause of bloodstream infections in AL patients. Carbapenem-resistant Enterobacteriaceae (CRE) is an emerging health problem worldwide; however, the incidence of CRE varies greatly between different regions. Carbapenem resistance in Enterobacteriaceae is caused by different mechanisms, and CRE may display various resistance profiles. Bacterial co-expression of genes conferring resistance to both broad-spectrum β-lactam antibiotics (including carbapenems) and other classes of antibiotics may give rise to multidrug-resistant organisms (MDROs). The spread of CRE represents a major treatment challenge for clinicians due to lack of randomized clinical trials (RCTs), a limited number of antibiotics available, and the side-effects associated with them. Most research concerning CRE infections in AL patients are limited to case reports and retrospective reviews. Current research recommends treatment with older antibiotics, such as polymyxins, fosfomycin, older aminoglycosides, and in some cases carbapenems. To prevent the spread of resistant microbes, it is of pivotal interest to implement antibiotic stewardship to reduce broad-spectrum antibiotic treatment, but without giving too narrow a treatment to neutropenic infected patients.

## 1. Introduction

Clinical manifestations of acute leukemia (AL) result either from the proliferation of leukemic cells or from bone marrow failure that leads to a decrease in normal cells; hence, AL patients usually present with pancytopenia, including neutropenia. In a classical study from 1966 [1], Bodey et al. studied the quantitative relationships between circulating leukocytes and infection in patients with acute leukemia. The study demonstrated that the risk of infections in granulocytopenic patients increased rapidly when granulocyte count dropped to <0.5 cells/µL and the majority of severe infections increased when the granulocyte count dropped to <0.1 × 10^6^ cells/µL.

Early identification of sepsis and microbiological diagnostics including cultures from blood and possible foci of infection, followed by prompt administration of intravenous antibiotics covering the most likely pathogens, remain the cornerstone in the initial management of sepsis. Until the microbiological etiology and susceptibility pattern of the infectious organism is known, choice of antibiotic treatment should be based on national or international guidelines, adjusted by knowledge of local epidemiology and susceptibility pattern. In addition, the patient’s individual risks of having infections with especially resistant bacteria must be considered. Increasing prevalence of carbapenem-resistant Enterobacteriaceae (CRE), often in combination with resistance to non-β-lactam antibiotics, considerably complicates both empirical and targeted treatment of infections in AL patients [2,3,4,5]. Lack of randomized clinical trials (RCTs) leaves the optimal treatment algorithm for CRE infections largely unknown. In addition, the coexistence of delayed and inadequate empirical antibiotic treatment is associated with high mortality and morbidity [6,7]. CRE includes primarily nosocomial agents, and CRE infections occur most frequently in institutionalized immunocompromised patients receiving medical care in hospitals, long-term acute care facilities, or nursing homes [8,9].

In this review, we address important issues regarding CRE infections in AL patients. However, most of the reviewed literature consists of retrospective small series and case reports. In addition, in a number of studies, AL patients constituted a subgroup of patients with hematological malignancies (HM). Only a limited number of studies focused on CRE infections in AL patients. We highlight epidemiology, risk factors, clinical implications, microbiological aspects, and diagnostics in AL patients with CRE infections. Lastly, we discuss future challenges and perspectives.

## 2. Methods

In this paper, we performed a nonsystematic review of carbapenem-resistant Enterobacteriaceae, with implications for treating acute leukemias, a subgroup of hematological malignancies. PubMed was searched using the following terms: [Date—Publication] AND [hematological malignancies] AND [carbapenem resistance] AND [Enterobacteriaceae]. The PubMed search returned 18 papers, of which nine papers were selected. Papers from January 2016, written in English, were included. One study of children was excluded. Abstracts and aims of the study were screened for relevance, excluding another six papers. All were screened for relevance to the subject of this review. Additional articles of interest, as well as selected series and case reports of interest that reported treatment, mortality, newer antibiotics, and colonization or infection of patients with CRE bacteria, were also included.

Carbapenemase-producing Enterobacteriaceae (CPE) denotes Enterobacteriaceae producing a carbapenemase (i.e., a β-lactamase able to hydrolyze carbapenems). Carbapenem-resistant Enterobacteriaceae (CRE) denotes phenotypic carbapenem-resistant Enterobacteriaceae, regardless of the mechanism, including both CPE and non-CPE CRE.

## 3. Acute Leukemia and Infection Risks

AL is a heterogeneous group of malignant hematopoietic diseases and is divided into two basic types: acute myeloid leukemia (AML) and acute lymphoblastic leukemia (ALL). The clinical presentation is often similar, with anemia and neutropenia caused by bone marrow failure due to severe thrombocytopenia [10,11]. Untreated patients die within weeks to months, and the only way of cure is intensive chemotherapy treatment, possibly in combination with hematopoietic stem-cell transplantation (HSCT) [10,11].

Infectious complications represent one of the main obstacles complicating the treatment of AL. In particular, there is a growing concern for multidrug-resistant organisms (MDROs) [12], including Enterobacteriaceae. Infection risk is related to the disease itself, other patient-related factors, and the treatment of the disease [12] (Table 1).

The combination of all these factors makes AL patients extra vulnerable and prone to infections. Noteworthy is also the differences in incidence and severity of infections and sepsis between AML and ALL patients, also reflected in differences in early mortality, often due to severe infections, which is significantly higher in AML patients compared to ALL patients [12].

During the 1960s and 1970s, Gram-negative bacteria (GNB) were the most frequent cause of infections in patients with HM. Since the mid-1980s, a shift toward Gram-positive bacteria (GPB) has been observed in many centers [13]. However, over the last few years, several centers have reported an increase in GNB bloodstream infections (BSIs) [14,15,16], whereas, in some centers, GNB are more frequently isolated than GPB [6,17,18,19]; however, there is a large variability between hospitals and countries [13,14].

Special for the setting of infectious complications is injury of mucosal barriers associated with leukemia treatment, which is the first line of defense against external pathogens [20]. Leukemia patients receiving cytotoxic therapy or radiotherapy experience mucosal barrier injury, often termed mucositis, creating an entrance point for resistant microorganisms, with the potential to cause BSIs [21]. In addition, the gastrointestinal microbiota is often affected during the treatment course of acute leukemia, through both mucosal barrier injuries and the use of broad-spectrum antimicrobial agents [21,22,23]. Use of broad-spectrum antibiotics increases the risk for colonization with MDROs, including CRE [24,25,26,27]. Intestinal MDRO colonization, previous use of antimicrobial therapy, especially broad-spectrum beta-lactams, and long-term hospitalization all increase the risk for BSI MDROs [27,28,29,30,31]. Emergence of CRE makes carbapenems a less secure choice in several parts of the world [27,32,33,34].

## 4. β-Lactam and Carbapenem Resistance in *Enterobacteriaceae*

β-lactamases are enzymes able to hydrolyze and, thus, inactivate β-lactam antibiotics. On the basis of amino-acid sequence similarity, β-lactamases are divided into four classes (A, B, C, and D) according to Ambler’s classification scheme [35], and each class is further subdivided into families. Substrate specificity (i.e., which β-lactam is hydrolyzed by the enzyme) ranges from penicillinases (hydrolysis of penicillins only), to cephalosporinases (hydrolysis of penicillin and cephalosporinases) and carbapenemases (hydrolysis of most/all β-lactams, including carbapenems) [36]. As single-point mutations may change the substrate specificity dramatically, enzymes within the same family may have very different substrate profiles. However, β-lactamases in the same family and class share some common properties (Table 2).

Bacteria may harbor genes encoding β-lactamases in their chromosome, rendering them naturally resistant to certain β-lactams [40]. However, genes encoding β-lactamases may also be incorporated into mobile genetic elements (transposons, plasmids), facilitating much more effective spread of the resistance determinants [41]. Plasmids may harbor several resistance determinants, conferring resistance to different classes of antibiotic, resulting in MDROs [42].

While wildtype *Esherichia coli* with no genes coding for β-lactamases is susceptible to most clinically relevant β-lactams, other Enterobacteriaceae species (e.g., all *Enterobacter* sp. and many *Citrobacter* sp.) are intrinsically resistant to penicillins and cephalosporins due to chromosomally encoded class C cephalosporinases (AmpC) [41]. Chromosomally encoded carbapenemases are uncommon in Enterobacteriaceae. However, these species may become carbapenem-resistant, either by producing a chromosomally or plasmid encoded cephalosporinase in combination with reduced influx and/or increased efflux of carbapenem or by acquisition of plasmids harboring genes encoding a carbapenemase [36]. Some very successful carbapenemase families (i.e., KPC, OXA, NDM, IMP, and VIM) have been transferred to mobile genetic elements and/or entered clinical successful Enterobacteriaceae clones and disseminated globally [43]. Some important characteristics of these carbapenemases are shown in Table 2. Even if the occurrence of carbapenemase-producing Enterobacteriaceae is a worldwide trend, there are great regional differences [36].

## 5. The Epidemiology of CRE Globally

In 2018, Brolund et al. [44] conducted an epidemiological study among 37 European nations. In 2018, all the participating countries reported CPE cases, whereas, in 2015, three countries were yet to identify a single case. Overall, 11 countries reported a higher epidemiological stage of CPE than in 2015, 25 countries described no change, and one country reported an improvement of the CPE epidemiological situation.

Righi et al. [45] conducted a systematic review of the global prevalence of carbapenem resistance in neutropenic patients between January 1995 and April 2016, including 30 studies from 21 countries. They reported overall carbapenem resistance ranging from 2–53% among studies. Infections due to carbapenem-resistant *Pseudomonas* spp. were reported in 60% of the studies. Resistance of Enterobacteriaceae was less common, and bloodstream infections due to carbapenem-resistant *Klebsiella* spp. were mainly documented from endemic areas (Greece, Italy, and Israel).

Data from the European Antimicrobial Resistance Surveillance Network (EARS-Net) showed significantly increasing trends for carbapenem resistance in *K. pneumoniae* in the European Union (EU)/European Economic Area (EEA) between 2015 and 2019 [46]. The annual increase during this period was more moderate compared to previous periods, with a range of carbapenem resistance in *K. pneumoniae* invasive isolates from 0–58% in 2019. Carbapenem resistance remained rare in *E. coli*, but there was a small yet significant increase between 2015 and 2019 [46].

In Norway, colonization and infections with CPE are notifiable conditions. The prevalence of CPE is low, but steadily rising. In a population of 5.2 million, 75 patients with CPE were identified in 2019. This is an increase from 34 cases in 2017 and 54 cases in 2018. Most CPE isolates belonged to globally disseminated clones associated with specific carbapenemase genes. Despite some small clusters, there is no clear evidence for interregional spread of CPE in Norway [47].

In an epidemiological study from Egypt 2020 [48], more than half of the hospitals (64%) had at least one CRE isolate. Nearly half (47.9%) of Enterobacteriaceae isolates were CRE, which is higher than estimates reported from other Arab, African, or Asian countries [49,50,51,52]. The incidence of CRE hospital-acquired infections (HAI) was also much higher than the overall incidence of all CRE (HAI and non-HAI) reported from other countries, including the United States, Canada, and China [53,54,55].

## 6. The Epidemiology of CRE in HM Patients

In 2020, Lalaoui et al. [56] reviewed series and selected case reports studying epidemiological and clinical infections due to carbapenem-resistant GNB in patients with HM [2,3,6,17,18,57,58,59,60,61,62,63,64,65,66]. In their study, resistance profiles of strains involved mainly reflected local epidemiology, and they observed that significant risk factors associated with infections with CRE included male, age around 50 years old, AL, selvage chemotherapy, neutropenia, and digestive tract colonization by carbapenem-resistant bacteria. They observed high mortality rates, particularly in AML patients with unresolved neutropenia, in patients treated with inappropriate empiric antibiotics, and in patients that received delayed administration of targeted antibiotics. These findings support the hypothesis that there is a relationship between the epidemiology of healthcare-associated infections and that found in HM patients.

In a study from two oncological centers in New York 2008–2012 [4], CRE was identified in 2.2% out of 1992 BSIs, and 4.7% of GNB. Independent risk factors for CRE BSI were prior β-lactam/β-lactamase inhibitor or carbapenem use, current trimethoprim-sulfamethoxazole or glucocorticoid use, and having a prior CRE-positive culture.

In a retrospective survey from Italy [57], epidemiology and prognostic factors of carbapenem-resistant *K. pneumonia* (CRKp) infections among auto- and allo-HSCT recipients in 52 transplant centers were assessed. The study showed a significant increase in the incidence of CRKp infection cases among allo-HSCT patients, from 0.4% in 2010 to 2.9% in 2013. Furthermore, 64% of the allo-HSCT patients had AL. The type of underlying disease among the auto-SCT patients was mainly represented by chronic lymphoproliferative malignancies, and the incidence of CRKp infections went from 0.1% in 2010 to 0.7% in 2013.

In Turkey, Kara Ali et al. [67] studied a cohort of hospitalized patients with HM and BSIs in the period between 2006 and 2016, of which Enterobacteriaceae accounted for 86% of the GNBs. From the first reported case in 2010, there was an increasing trend in carbapenem resistance among Enterobacteriaceae, which was 6.5% in *E. coli* and 32.8% in *Klebsiella* spp. at the end of the study period.

Kara et al. [18] performed a retrospective review study focusing on the epidemiology and the emergence of resistance in BSIs in patients with HM in Turkey between 2005 and 2009. GNB were the predominant pathogens isolated among the cases of BSIs. The patients were divided into high-risk or low-risk groups according to their tendency to develop severe infections. ALs (AML 43.2% and ALL 34.8%) were the most common diagnosis in patients with high-risk HM. Moreover, 10% of *K. pneumonia* isolates in the high-risk group were resistant to meropenem, while none of the *K. pneumoniae* isolates in the low-risk group were carbapenem-resistant. There was no carbapenem resistance in *E. coli* in any of the patient groups.

## 7. CRE: Clinical Aspects

Infections caused by CRE are associated with high morbidity and mortality rates, and patients admitted to the intensive care unit (ICU) or patients with serious underlying conditions are at a high risk. This makes CRE a public health problem, causing hospital outbreaks worldwide [68].

*Enterobacteriaceae* can easily spread between immunocompromised institutionalized patients and staff through hand carriage, contaminated food and water, and acquired genetic material through horizontal gene transfer mediated mostly by plasmids and transposons [69]. The isolates that produce carbapenemase are usually also resistant to many non-β-lactam classes of antibiotics such as fluoroquinolones, aminoglycosides, and co-trimoxazole [69,70,71].

Most CRE infections occur in hospitals and long-term care facilities, and these represent the primary transmission environments for CRE infections. Previous reports have documented residence in a long-term care facility, admission to intensive care units, and hospital care as risk factors for acquiring CRE infections [5,68]. Among patients found to be infected with CRKp and carbapenem-resistant *Acinetobacter baumannii*, 75% and 51.3% were admitted from post-acute care facilities, respectively. Additionally, 75% of the patients had repeated hospital admissions within the previous year [72]. Sources associated with CRE infections within hospitals are sinks, patient beds, and mechanical ventilation equipment as significant risk factors [68]. Other risk factors associated with CRE infections were previous exposure of antimicrobials like carbapenems, cephalosporins, glycopeptides, quinolones, and beta-lactams [68,73].

Most previous reviews on CRE have focused on hospital acquired infections (HAIs); however, due to the highly transmissible nature of plasmid-borne carbapenemases, reports have warned about the likely spread into the community from healthcare settings [74,75,76,77,78,79,80].

Kelly et al. [81] performed a review of the literature to assess the percentage of CRE isolates that could be associated with the community. Fifteen studies were included, of which five studies found no community associated CRE. However, 10 studies identified percentages ranging from 0.04–29.5% of either community-associated or community-onset cases among their samples, with United States (US)-based studies alone ranging from 5.6–10.8% [53,55,82,83,84,85,86,87,88,89].

CRE colonizing strains can go on to cause infections or spread to other patients. Since hospital settings are the primary route of transmission, identification and containment of these bacteria represent an important screening intervention [57,90,91]. The intestinal microbiota contains Enterobacteriaceae, and rectal swab or stool is the preferred specimen for detection of CRE [90,92]. In general, CRE colonization is associated with a higher chance of developing BSIs caused by the same bacteria [13]. In a review by Tischendorf et al. [90], they assessed the risk of CRE infection following colonization with CRE. They included a total of 1806 hospitalized patients colonized with CRE, where 16.5% developed clinical infections. AL patients are at a high risk of developing CRE colonization, and both the underlying hematological disease and the neutropenia are associated with BSI development in CRE-colonized patients [58,62,93]. In addition, the increased risk of developing invasive CRE infections in patients with HM has been associated with risk factors such as treatment-induced neutropenia, prolonged hospital stays, frequent use of broad-spectrum antibacterial agents and gastrointestinal mucositis [94,95]. In the general population, CRE most commonly causes urinary tract infections and pneumonia, and indwelling medical devices present a high risk of acquiring CRE infections [68,72,82,96,97,98,99]. In an Italian multicenter study [63], the mortality rate among patients with HM with CRKp BSI was 52.2%. Other studies have reported mortality rates of CRKp bacteremia in HM patients ranging from 53–57.6% [64,100]. The proportion of AML patients in these studies was 53.8% and 57%, respectively.

## 8. Detection of CRE and Characterizing of Carbapenemases

CRE may be detected in clinical samples by culture or by nucleic acid amplification technologies (NAATs) [101,102]. Culture-based methods detect both CPE and non-carbapenemase-producing-CRE (non-CP-CRE), while NAATs detect only selected carbapenemase genes [102]. However, time of detection of CRE by culture-based methods alone may be several days, compared to minutes/hours (analytic time) by NAATs. Commercially available NAATs for direct detection of CPE in clinical samples differ, among others, in assay coverage (which carbapenemase genes are detected), workflow, hands-on time, time to result, and costs [103]. As many of the NAATs also are validated for use on positive blood cultures and bacterial colonies, a mixture of culture-based methods and NAATs is possible, e.g., by ruling in or out the presence of specific carbapenemase genes in a positive blood culture or in a phenotypic CRE isolate. There are also several phenotypic methods available to detect and characterize carbapenemases in positive blood cultures and/or in CRE isolates. These include growth-based techniques (modified Hodge test, carbapenem inactivation methods, targeted carbapenemase assays), lateral flow immunoassays, biochemical assays detecting in vitro carbapenemase activity in bacterial extract (Carba NP test and variants), and MALDI-ToF MS [101,102].

## 9. Management of CRE Infections in Adult Acute Leukemia Patients

Early identification of sepsis, followed by diagnostic blood cultures, obtaining source control, and prompt administration of appropriate intravenous antibiotics, remains the cornerstone in initial management [104]. The coexistence of delays and inadequacy of treatment is associated with lower survival rates. Therefore, the 2016 surviving sepsis guidelines recommend that the initial empiric antibiotic treatment should include a broad-spectrum antibiotic (alone or in combination) that has activity against the most likely pathogens and should be initiated within 1 h [104].

There is a limited selection of antibiotic options available for treatment of CRE infections, and most antibiotic classes available are associated with notable side-effects [5]. Antibiotic classes available and proven effective in the treatment of CRE infections in adult HM patients include colistin, tigecycline, and gentamicin, with at least one of them active in vitro against the isolate [57,105]. The standard therapy required in HM patients with documented CR bacterial infection is one of the following combinations: tigecycline + gentamicin, colistin + gentamicin, colistin + tigecycline + gentamicin, or colistin + tigecycline.

Globally, CRE strains demonstrate susceptibility to colistin in 80–100%, tigecycline in 60–80%, and gentamicin in 10–60% of HM patients [56]. The highest level of aminoglycoside resistance was observed in India [58] and Italy [62], where CRKp isolates with a high minimum inhibitory concentration (MIC) to gentamicin, tigecycline, and colistin were reported [106]. In a study from Italy, Micozzi et al. [62] observed that, among the 22 *K. pneumonia* isolates registered, only 53.5% of the isolates were susceptible to colistin, 27% were susceptible to tigecycline, and none were susceptible to gentamicin. In previous studies, only 34.1%, 24% and 14% of HM patients with CRE infections in Israel, Italy, and the US, respectively, received an adequate antibacterial regimen [2,4,57].

For high-risk neutropenic patients, most guidelines recommend starting monotherapy with a beta-lactam active against *P. aeruginosa* (piperacillin-tazobactam, imipenem, meropenem, cefepime, or ceftazidime) and adding a second antibiotic in clinically unstable patients with increased risk of MDR bacteria [105]. In light of the increasing frequency of ESBL and CRE, choosing broad-spectrum empiric antibiotics covering MDR bacteria must be considered. In a retrospective study at 22 centers from nine different countries (Argentina, Australia, Brazil, Canada, Germany, Italy, Spain, Turkey, and the United States) from 1 January 2006 to 31 March 2015, β-lactam/β-lactamase inhibitors were compared to carbapenems in two cohorts of hematological neutropenic patients with extended-spectrum β-lactamase BSI: the empirical therapy cohort (174 patients) and the definitive therapy cohort (251 patients). They found that 30 day case fatality rates and other secondary outcomes were similar in the two therapy groups of the two cohorts and also in the propensity-matched cohorts. They concluded that β-lactam/β-lactamase inhibitors might be carbapenem-sparing alternatives for the treatment of BSI due to extended-spectrum β-lactamases in these patients [107]. Although the frequency of CRE infections is emerging, RCTs in septic AL patients with CRE infections have not been implemented. Therefore, most existing evidence is from reviews of case reports, case series, and small retrospective studies, which have a number of inherent limitations [108,109].

**Figure 1:**
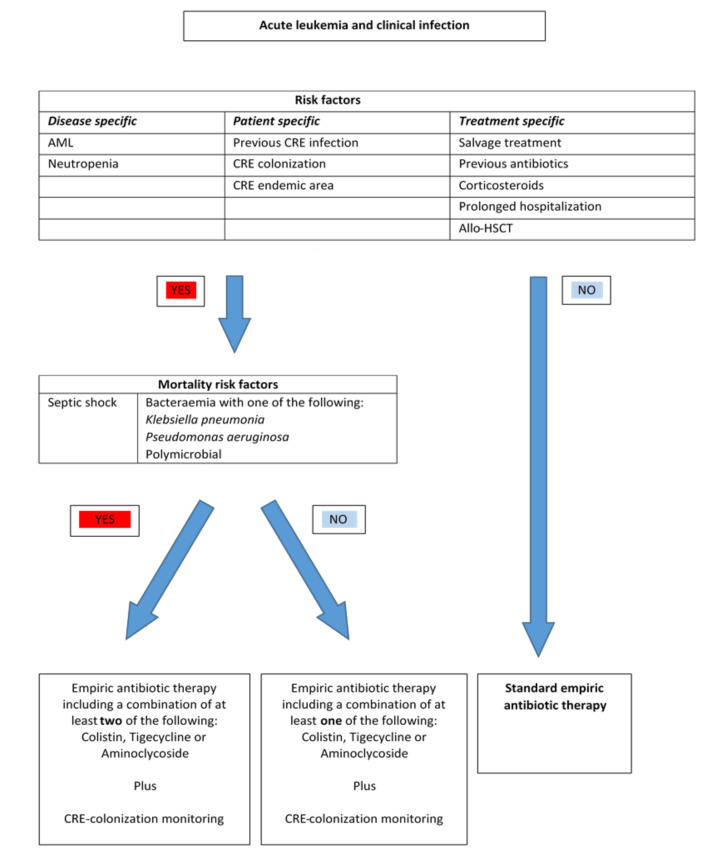
An algorithm of risk assessment and empirical management of febrile patients with hematological malignancies (HM). Based on Lalaoui [56].

The first-line treatments of carbapenem resistant bacteria do not correspond to standard empiric antimicrobial treatment recommended in HM patients. Resistance profiles in HM patients often reflect local epidemiology [56]. Therefore, the therapeutic strategies in febrile HM patients should be based on early identification of increased risk for carbapenem-resistant bacterial infections and local antimicrobial susceptibility profiles. Figure 1 is based on an algorithm proposed by Lalaoui et al. [56]. The figure shows recommended empirical management guided by risk assessment of factors related to morbidity and mortality due to infections caused by carbapenem-resistant bacteria in febrile HM patients.

In a comprehensive study by Falagas et al. [110], they sought to evaluate the effectiveness of the antibiotic treatment administered for infections caused by CRE. Three studies mention an important survival benefit combining a carbapenem with another antibiotic [111,112,113]. According to the authors, in one out of three studies [113], this could be explained by the fact that meropenem was the antibiotic most commonly added to the colistin + tigecycline combination. In addition, they observed increased survival in patients infected with isolates for which the meropenem MIC was ≤4. This observation is supported by pharmacokinetic/pharmacodynamics data [114] and in accordance with the clinical breakpoints set by the European Committee on Antimicrobial Susceptibility Testing (EUCAST) [115].

A limited number of reports also documented successful results using a double-carbapenem regimen [116,117]. In 2016, Oliva et al. [118] reported 15 patients with CRE infections treated with a combination of two carbapenems, reporting an 80% response rate. The rationale for this treatment strategy is that KPC has greater affinity for ertapenem than other carbapenems. The binding of ertapenem hinders doripenem/meropenem degradation in the environment of the microorganism [119].

New drugs such as ceftolozan/tazobactam, meropenem/vaborbactam, and ceftazidime/avibactam should also be considered, following susceptibility testing. Although new antibiotics have shown promising results in clinical trials, there is still uncertainty over whether their use will improve clinical outcomes in real-world practice [120,121]. There is a lack of RCTs focusing specifically on treating infections caused by CR GNB with ceftazidime/avibactam. Experience from studies comparing treatment with ceftazidime/avibactam to other regimens in the treatment of severe infections caused by CRE has been associated with higher rates of clinical success and survival [122,123,124]. A point of specific interest is the broad inhibitory profile of avibactam (inhibits class A, B, and D β-lactamases (Table 2), including many of the most common carbapenemases in these classes). As many MDR isolates harbor several different β-lactamases from different classes, ceftazidime/avibactam may be an option for Enterobacteriaceae resistant to other β-lactams, including carbapenems (Table 2).

In vitro synergy between polymyxins and carbapenem has been demonstrated in several studies, despite resistance to carbapenems alone [125,126]. Treatment is centered on a polymyxin backbone coupled with other targeted antibiotics. Meta-analyses of different antibiotic strategies demonstrated a lower mortality rate when using polymyxin-based treatment regimens, as well as a triple combination of polymyxin, carbapenem, and rifampin or tigecycline [127]. Combination therapy with polymyxins and other relevant antibiotics has demonstrated synergistic effects, as well as bactericidal properties. Therefore, combination therapy should be considered [5,125,126,128].

Treatment of infected AL patients remains a major challenge due to a limited number of studies [2,57,58,60], and RCTs focusing solely on AL patients do not exist. Many studies of neutropenic patients have included few patients or have not performed multivariate analyses. Most data on treatment of ESBL-producing Enterobacteriaceae come from case series and observational studies in non-neutropenic patients [129].

In a study by Tzouvelekis et al. [109], reviewing 20 clinical studies, they reported that treatment with a single in vitro active antibiotic resulted in mortality rates not significantly different from those observed in patients treated with no active therapy, whereas combination therapy with two or more in vitro active agents was superior to monotherapy. The lowest mortality rate was observed in patients treated with carbapenem-containing combinations. Potential advantages of combination therapy include improved efficacy due to synergy.

## 10. Future Challenges and Perspective

CRE is an emerging global health problem and represents a major threat to immunocompromised patients. CRE is associated with high mortality rates and few therapeutic options, due to the ability to confer resistance to many different classes of antibiotics. To prevent vulnerable immunocompromised hosts from becoming infected with CRE, a joint effort between the scientific and medical community is necessary. Empirical treatment with a combination of antibiotics should be started at the onset of fever rather than after identification of the actual pathogen. Nevertheless, several studies have documented that the coexistence of delayed and inadequate empirical antibiotic treatment occurs. Therefore, the logical step to control the spread of CRE is to take measures to prevent immunocompromised institutionalized patients from becoming carriers and infected with CRE. Patients who are carriers of or have clinical infections with CRE can act as reservoirs for transmission to other patients, resulting in carriage, infection, or outbreaks. CRE infections are so far mainly an institutional problem. However, to prevent CRE from spreading within long-term facilities and hospitals to the community, it is of pivotal interest to know the local CRE epidemiology to make the best treatment decisions.

Strategies to prevent CRE infections include implementation of contact precautions to reduce transmission of CRE, in addition to aggressive environmental cleaning to achieve infection control. Surveillance to identify CRE carriers in healthcare settings and early identification of new cases are recommended in outbreak scenarios in endemic regions. Strengthening of microbiological services includes using optimized, fast, and sensitive methods to detect infections and CRE carriers, and, when positive results are available, the results should be communicated to the relevant healthcare staff. Antibiotic stewardship is also important, especially in clinical scenarios where clinical signs are scarce, and where fever may often be the only sign of ongoing infection. Nevertheless, clinicians should strive to optimize the antibacterial dosages, assess daily the need for continued antibacterial therapy, and de-escalate the use of broad-spectrum antibiotics in stable patients when possible, to limit the unnecessary use of broad-spectrum antibiotics.

Lastly, there is an urgent need for RCTs to determine optimal empirical and target treatment algorithms for different patient groups in different countries and continents.

## Figures and Tables

**Table 1:** Infectious risk in acute leukemia patients. Main factors are related to infection risk in leukemia patients.

**Disease-specific factors**	Quantitative and qualitative defect of neutrophil granulocytes Reduced humoral immunity Reduced immune-mediated B-lymphocyte effects Reduced immune-mediated T-lymphocyte effects
**Patient-related factors**	Age Comorbidity conditions Malnutrition Socioeconomical status
**Treatment-related factors**	Disruption of mucosal barrier Gastrointestinal bacterial colonization Previous antibiotic treatments Central venous catheter or other devices

**Table 2:** Effect on different β-lactams and inhibitor activities against a selection of β-lactamase-families. Based on data from [37,38,39].

		Ambler Class									
		β-Lactamase Family (Examples)									
		Class A				Class B			Class C	Class D	
		TEM-ESBL	SHV-ESBL	CTX-M	KPC *	IMP *	VIM *	NDM *	CMY	OXA-1	OXA-48 *
Degrading	Temocillin	−	−	−	+	++	++	++	−	−	++
	Ceftfazidime	++	++	++	++	++	++	++	++	−	−
	Aztreonam	++	++	++	++	−	−	−	++	−	−
Inhibited by	Clavulanate	++	++	++	−	−	−	−	−	+	−
	Sulbactam	++	++	++	−	−	−	−	−	+	−
	Tazobactam	++	++	++	−	−	−	−	+/−	+	−
	Avibactam	++	++	++	++ **	−	−	−	++	++	++
	Relebactam	++	++	++	++	−	−	−	++	+	−
	Vaborbactam	+	+	++	++	−	−	−	+		−

* Carbapenemases; ** not reliable with KPC−3; − not degraded/no inhibition; + partly degraded/ weak inhibition; ++ degraded/inhibition; +/− variable.

## Data Availability

Not applicable.
